# Efficacy and safety of Zhibitai in treating coronary heart disease patients with hyperlipemia: A protocol for systematic review and meta-analysis

**DOI:** 10.1097/MD.0000000000021991

**Published:** 2020-09-04

**Authors:** Mingtai Chen, Ying Li, Ling Men, Zhong Zhang, Xiaoling Zhong, Shudong Yang, Jienan Luan

**Affiliations:** a Department of Cardiovascular Disease; b Nephrology Department; c Reproductive Health Department, Shenzhen Traditional Chinese Medicine Hospital, Shenzhen, China.

**Keywords:** coronary heart disease, meta-analysis, protocol, randomized trial, Zhibitai

## Abstract

**Objective:**

: To assess the clinical efficacy and safety of Zhibitai, as a kind of natural lipid-lowering Chinese medicine, in treating coronary heart disease patients with hyperlipemia

**Methods:**

: A systematic literature search for articles up to July 2020 will be performed in following electronic databases: PubMed, Embase, the Cochrane Library, China National Knowledge Infrastructure, Chinese Scientific Journals Database Database, Chinese Biomedical Database, Chinese Biomedical Literature Service System, and Wanfang Database. Inclusion criteria are randomized controlled trials of Zhibitai applied on coronary heart disease patients with hyperlipemia. The primary outcome measures will be serum lipid levels (including total cholesterol, triglyceride, low-density lipoprotein cholesterol, and high-density lipoprotein cholesterol) and acute cardiovascular events. The secondary outcome measures will be inflammatory factors (high sensitive C-reactive protein, interleukin-6, etc), safety index (liver function, renal function, etc), and adverse events. RevMan 5.3 software will be used for data synthesis, sensitivity analysis, meta-regression, subgroup analysis, and risk of bias assessment. A funnel plot will be developed to evaluate reporting bias and Egger tests will be used to assess funnel plot symmetries. We will use the grading of recommendations assessment, development, and evaluation system to assess the quality of evidence.

Trial registration number PROSPERO CRD42018103341.

## 1 Introduction

Hyperlipemia, including elevated low-density lipoprotein cholesterol (LDL-C) or total cholesterol (TC) or triglyceride (TG) or low high-density lipoprotein cholesterol (HDL-C), has played a significant role in the development and progression of atherosclerosis.^[1–2]^ Atherosclerosis has been considered as an inflammatory reaction of blood vessels combined with lipid accumulation, which contributed to the formation of the atherosclerotic plaque leading to subsequent acute cardiovascular events.^[3]^ Therefore, hyperlipemia has been regarded as a critical risk factor for coronary heart disease (CHD).^[4]^

Statins, a significant therapeutic strategy of lipid lowering, have been the necessary treatment for CHD patients or primary prevention of cardiovascular diseases in high-risk populations.^[5]^ A growing amount of evidence indicated that decreased incidence of all-cause mortality and acute cardiovascular events through statins treatment in CHD patients were strongly associated with the lipid lowering and anti-inflammatory effects of statins.^[6,7]^ In spite of the great benefit of statins treatment, the increasing risk of adverse effects (such as myopathy, hepatic toxicity, hyperglycemia, and impaired steroid production) of statins due to the higher doses could not be ignored.^[8]^

Zhibitai, a kind of Chinese patent medicine, is comprised of a family of naturally occurring statins, which has been widely used in China. Because of its efficacy of lipid-lowering and low incidence of adverse events, it has been recommended by Chinese expert consensus on the use of Zhibitai capsules in 2017 and becoming more and more widely applied for hyperlipemia in China.^[9]^ However, the intensity of evidence has been poor and there has been lack of systematic analysis to assess the efficacy and safety of Zhibitai for the treatment of CHD with hyperlipemia from randomized controlled trials (RCTs).^[10]^ Therefore, in consideration of the insufficient and limited evidences of widespread use of Zhibitai, this study aims to evaluate objectively the efficacy and safety of Zhibitai for CHD patients with hyperlipemia by integrating the existing trials.

## 2 Methods and analysis

### 2.1 Registration

The study protocol has been registered on international prospective register of systematic review (PROSPERO). The trial registration number of prospective register of systematic review is CRD42018103341. The procedure of this protocol will be conducted according to the preferred reporting item for systematic review and meta-analysis protocols guidance.^[11]^

### 2.2 Eligibility criteria

#### 2.2.1 Type of study

We will include all the RCT s that investigated the efficacy and safety of Zhibitai capsule/decoction combined with pharmacotherapy for the treatment of CHD with hyperlipemia. The studies will be excluded if it is not an RCT (namely, observational cohort and case–control studies, case reports, experimental studies, and reviews).

#### 2.2.2 Participants

The study will include patients diagnosed as CHD with hyperlipemia regardless of their age, sex, ethnicity, education or economic status and whether or not they were outpatients or inpatients. The diagnostic criteria of CHD and hyperlipemia are as follows.

The diagnostic criteria of CHD should be confirmed according to one of the past or current definitions. The diagnostic criterias include Report of the joint International Society and Federation of Cardiology/World Health Organization task force on standardization of clinical nomenclature of ischemic heart disease or American College of Cardiology/American Heart Association guideline update for the management of patients with chronic stable angina or Chinese Association of Cardiology or Unstable angina pectoris diagnosis and treatment recommendations.^[12–14]^

The diagnostic criteria of hyperlipemia should be confirmed according to one of the past or current definitions. 2016 European Society of Cardiology/European Atherosclerosis Society Guidelines for the management of dyslipidaemias^[15]^ or the guidelines for prevention and treatment of hyperlipemia in Chinese adults (Revised Edition 2016).^[16]^

### 2.3 Interventions

Interventions involving the combination of Zhibitai with conventional pharmacotherapy are eligible in intervention group. The same conventional pharmacotherapy must be used in the control group.

### 2.4 Outcome

The primary outcome measures will include: Serum lipid levels (including TC, TG, LDL-C, and HDL-C) and acute cardio-cerebrovascular events. The secondary outcome measures will include: inflammation marker (high sensitive C-reactive protein, interleukin-6, etc) and adverse events.

### 2.5 Search strategy

The following electronic bibliographic databases will be searched from inception to July 2020: PubMed, Embase, the Cochrane Library, China National Knowledge Infrastructure, Chinese Scientific Journals Database Database, Chinese Biomedical Database, Chinese Biomedical Literature Service System, and Wanfang Database. There are no limits on the language of publication. Only clinical trials as a limitation will be included and searched. The following sources will also be searched to identify clinical trials which are in progress or completed: Clinical Trials.gov and the World Health Organization clinical trials registry. The additional relevant studies will also be retrieved from the reference lists of systematic reviews and included studies. We will map search terms to controlled vocabulary if possible. In addition, the search strategy for selecting the fields of title, abstract or keyword will be different referring to the characteristics of databases. Search terms are grouped into 3 blocks (see Table 1).

**Table 1:**
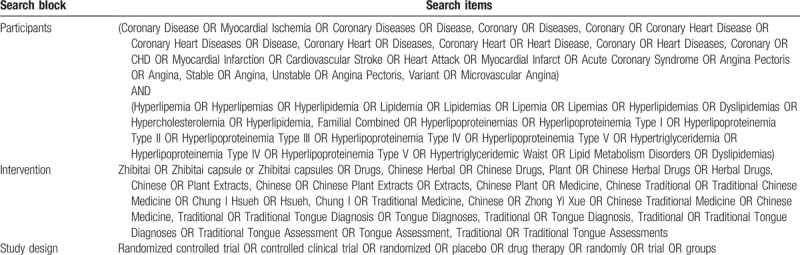
Search items.

### 2.6 Study selection and data extraction

**Figure 1:**
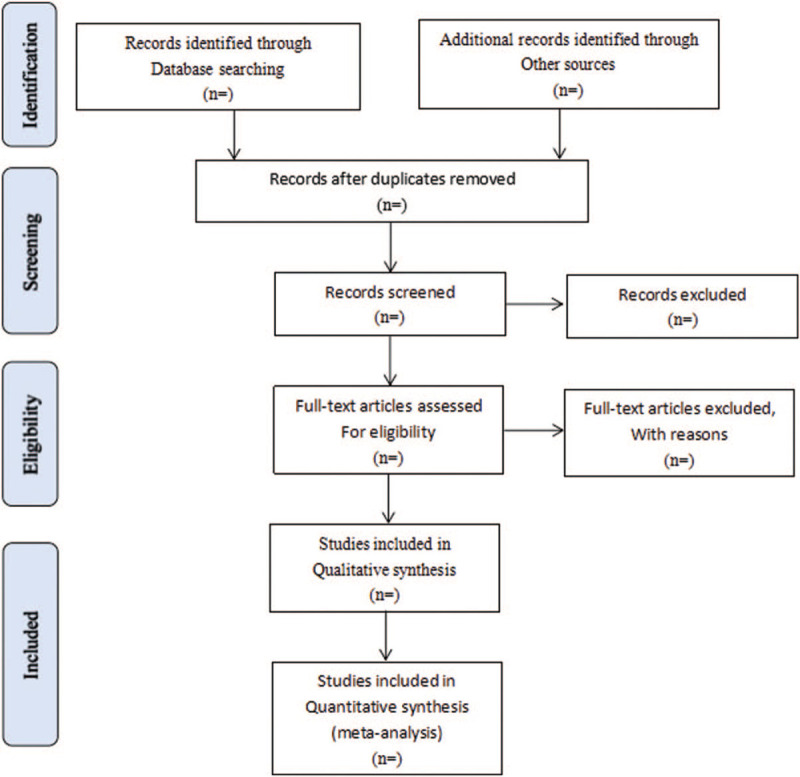
Flow diagram of study selection process. PubMed, Embase, the Cochrane Library, CNKI, VIP Database, CBM, SinoMed, and Wanfang Database. CBM = Chinese Biomedical Database, CNKI = China National Knowledge Infrastructure, SinoMed = Chinese Biomedical Literature Service System, VIP = Chinese Scientific Journals Database.

Literature retrieved citations will be managed by EndNote X7 software. Two authors (MC and LM) will screen the titles and abstracts of the all studies retrieved in above electronic databases independently to find potentially eligible studies. Articles which are duplicated or not accordant with eligibility criteria, intervention, and outcome in this study will be exclude. After filtering the final eligible articles, the data from the included articles will be extracted independently from 2 authors (MC and YL). Disagreements will be resolved by discussion or arbitrated by a third author if needed. The following data items will be extracted: first author, publication year, diagnose information, age, sex, trial characteristics, interventions and controls, participants, study methodology, outcomes, adverse events, and so on (see Fig. 1).

### 2.7 Risk of bias assessment

The methodological quality of the eligible studies will be evaluated according to the Cochrane Collaboration's tool for assessing risk of bias.^[17]^ The assessment details include: sequence generation, allocation concealment, blinding of participants and personnel, blinding of outcome assessors, incomplete outcome data, selective reporting, and other sources of bias. Each domain will be assessed as “low risk” or “high risk” or “unclear risk” according to the description details of eligible studies.

### 2.8 Data synthesis and statistical analysis

Statistical analyses will be conducted with RevMan 5.3 software provided by Cochrane Collaboration. Data will be presented by risk ratio or odd ratio with its 95% confidence interval for dichotomous outcomes and standardized mean difference or weighted mean difference with its 95% confidence interval for continuous outcomes. The *I*^2^ test will be calculated to determine the amount of heterogeneity. The results of the studies could be used the fixed-effect model to combined unless *I*^2^ statistic is more than 50%, in which cases, the random-effects model will be used.

### 2.9 Sensitivity analysis, subgroup analysis, and meta-regression

If the heterogeneity or inconsistency among the studies was detected, sensitivity analysis or subgroup analysis or meta-regression analysis will be performed. Subgroup analysis will be conducted to explore potential sources of heterogeneity according to the characteristics of studies, including sample size, severity of CHD, dose of Zhibitai, treatment duration, and other relevant parameters. If data extraction is insufficient, we will create a qualitative synthesis.

### 2.10 Publication bias

A funnel plot will be developed to evaluate reporting bias of the included studies. We will use Egger tests to assess funnel plot symmetry and will interpret values of *P* < .1 as showing statistical significance.

### 2.11 Quality of evidence

We will also assess the quality of evidence for the main outcomes with the grading of recommendations assessment, development, and evaluation approach. The 5 items will be investigated, including limitations in study design, inconsistency, inaccuracies, indirectness, and publication bias.

### 2.12 Patient and public involvement

Patients and/or public will not involve due to this study belonging to the secondary sources analysis.

## 3 Discussion

Hyperlipemia has been an important factor and an independent risk factor of CHD. Consequently, the therapeutic strategy of lipid lowering has contributed great significance to treatment of CHD.^[18]^ Statins have remained the main lipid lowering treatment in current.^[6–7]^ However, the adverse effects of statins, including myopathy, hepatic toxicity, and so on, have limited the long-term application of statins.^[8]^ Fortunately, Zhibitai which is composed of 4 different Chinese medicines (hawthorn, atractylodes rhizome, alismatis rhizoma, and, Monascus spp. [also named Red yeast rice extract]) might provide an alternative and complementary therapy for CHD patients with hyperlipemia by lowering lipid. Among the components mentioned above, Monascus spp., a natural plant lovastatin acid, can inhibit HMG-CoA reductase. Hawthorn aids digestion, regulates blood lipoprotein, improves antioxidant, and regulates immune function. Atractylodes rhizome decreases LDL-C and anti-platelet aggregation.^[19]^ Furthermore, increasing number of studies reported that Zhibitai has been widely used and of great benefit in improving CHD patients with hyperlipemia by lowering LDL, TC, TG, upregulating HDL and downregulating inflammatory factors. In spite of this, there has been no complete evaluation of the clinical evidence regarding Zhibitai as intervention for CHD patients with hyperlipemia in evidence-based medicine.^[20–22]^

Accordingly, we aim to conduct this systematic review to assess the efficacy and safety of Zhibitai for CHD patients with hyperlipemia. The results of this systematic review may help to propose the clinical recommendation for CHD patients with hyperlipemia and to provide more reliable evidence for Zhibitai application.

## Author contributions

**Conceptualization:** Zhong Zhang.

**Data curation:** Ying Li.

**Formal analysis:** Ying Li.

**Funding acquisition:** Zhong Zhang.

**Investigation:** Mingtai Chen.

**Methodology:** Mingtai Chen.

**Project administration:** Xiaoling Zhong, Jienan Luan.

**Resources:** Ling Men, Xiaoling Zhong.

**Software:** Ling Men.

**Supervision:** Xiaoling Zhong, Shudong Yang, Jienan Luan.

**Validation:** Shudong Yang.

**Visualization:** Shudong Yang.

**Writing – original draft:** Mingtai Chen.

**Writing – review & editing:** Mingtai Chen.
